# Analysis of the Stress and Displacement Distribution of Inferior Tibiofibular Syndesmosis Injuries Repaired with Screw Fixation: A Finite Element Study

**DOI:** 10.1371/journal.pone.0080236

**Published:** 2013-12-03

**Authors:** Qinghua Liu, Kun Zhang, Yan Zhuang, Zhong Li, Bin Yu, Guoxian Pei

**Affiliations:** 1 Department of Orthopaedic Trauma, Hong-Hui Hospital, Xi'an Jiaotong University College of Medicine, Xi'an, China; 2 Department of Orthopaedic Trauma, Nanfang Hospital, Southern Medical University, Guangzhou, China; 3 Xijing Hospital, The Fourth Military Medical University, Xi'an, China; Georgia Regents University, United States of America

## Abstract

**Background:**

Studies of syndesmosis injuries have concentrated on cadaver models. However, they are unable to obtain exact data regarding the stress and displacement distribution of various tissues, and it is difficult to compare models. We investigated the biomechanical effects of inferior tibiofibular syndesmosis injuries (ITSIs) and screw fixation on the ankle using the finite element (FE) method.

**Methodology/Principal Findings:**

A three-dimensional model of a healthy ankle complex was developed using computed tomography (CT) images. We established models of an ITSI and of screw fixation at the plane 2.5 cm above and parallel to the tibiotalar joint surface of the injured syndesmosis. Simulated loads were applied under three conditions: neutral position with single-foot standing and internal and external rotation of the ankle. ITSI reduced contact forces between the talus and fibula, helped periarticular ankle ligaments withstand more load-resisting movement, and increased the magnitude of displacement at the lower extreme of the tibia and fibula. ITSI fixation with a syndesmotic screw reduced contact forces in all joints, decreased the magnitude of displacement at the lower extreme of the tibia and fibula, and increased crural interosseous membrane stress.

**Conclusions/significance:**

Severe syndesmosis injuries cause stress and displacement distribution of the ankle to change multidirectional ankle instability and should be treated by internal fixation. Though the transverse syndesmotic screw effectively stabilizes syndesmotic diastasis, it also changes stress distribution around the ankle and decreases the joint's range of motion (ROM). Therefore, fixation should not be performed for a long period of time because it is not physiologically suitable for the ankle joint.

## Introduction

The inferior tibiofibular syndesmosis is one of the most important parts for maintaining the structural stability of the ankle mortise during weight transmission and walking [1]–[3]. While the inferior tibiofibular and talocrural joints share geographic proximity, injuries to these articulations are quite distinct. Injury to the syndesmotic tibiofibular joint are much less prevalent than lateral ankle sprains, but recognition has increased in recent years due to a heightened awareness of its mechanism, symptoms, signs of injury, and poorer outcomes [4]. Its injuries are reported to account for about 1–11% of all ankle injuries [5]–[8]. However, it may be considerably higher in populations involved in sporting activities [9]–[11]. The latest statistics show that between 17% and 74% of ankle injuries among young athletes, especially skiers and football, soccer, and hockey players, are inferior tibiofibular syndesmosis injuries (ITSIs) [9], [12]–[13]. Although ITSIs are mostly seen in combination with ankle fractures, they can also be isolated [2]. The primary role of the syndesmosis is to maintain congruency of the tibiotalar interface under physiologic axial loads. Recognition of the subtle anatomical changes inherent to this pathology allows prompt attention to the significant impairments known to delay functional recovery following this injury [4].

The biomechanical mechanisms of ITSIs and how to address this injury have been a subject of controversy. Although many researchers [3], [14]–[17] have performed numerous biomechanical studies in cadaveric specimens and proposed a number of suggestions, traditional research methods cannot elucidate stress distribution or the displacement transmission mechanism at the interior of the ankle and are easily influenced by many factors that make it difficult to compare findings among studies. Furthermore, they are often costly, time-consuming, and inefficient. To solve these problems, more and more researchers have turned to methods that employ digital technology and numerical analysis. Computational models greatly improve the predictive ability to acquire information about the mechanical mechanisms of the human body's inner structure that may not be easily obtained in experimental studies.

The purpose of this study was to investigate the biomechanical effect of ITSI and screw fixation of the ankle by finite element (FE) analysis. These biomechanical data may provide a theoretical reference for clinical treatment of ankle injuries.

## Materials and Methods

The geometrically accurate FE model was obtained from the three-dimensional (3D) reconstruction of CT images of the right foot of a normal male volunteer (30 years old, 172 cm in height, and 60 kg in weight) in the neutral unloaded position, whose foot had no history of trauma and no abnormality by X-ray examination. Cross-sectional CT images were taken with 0.625-mm intervals from the plane 20 cm above the ankle down to the plantar surface. Prior to CT scanning, the volunteer was informed of the experimental procedures and provided written informed consent. The study was approved by the Medical Research Ethics Committee of Hong-Hui Hospital, Xi'an Jiaotong University College of Medicine and was performed in accordance with the Declaration of Helsinki.

In this study, highly detailed boundary surfaces were fit to every bone using several automated and manual techniques available in Mimics (Materialise, Leuven, Belgium). For example, thresholding segmentation and region growing tools were used to reconstruct the 3D structure of each bone. Then, the data were exported in point cloud file format and transferred into SolidWorks 2009 (SolidWorks Corporation, Concord, MA, USA), using the guide of grid processing and surface generation to form geometric models. Next, the separate solid objects representing the bones were assembled within SolidWorks to form the foot/ankle complex. Then, a five-bone assembly 3D model of the ankle was established including the tibia, fibula, talus, calcaneus, and navicular. The coordinate axes of the assembly were aligned so that the X-axis pointed medially, the Y-axis pointed posteriorly (toe to heel), the XY plane paralleled the sole, and the Z-axis pointed upward (heel to knee). Finally, the data were imported into the Simulation module of SolidWorks to establish a FE model of the ankle. The simulation allowed for the application of 3D contacts, springs, forces, and torques.

A new static example was created based on the above assembly in Simulation. The bony structures were idealized as homogeneous, isotropic, and linearly elastic, with the Young's modulus and Poisson's ratio assigned as 7300 MPa and 0.3, respectively, according to the model developed by Gefen et al. [18]. These parameters were selected by weighing cortical and trabecular elasticity values [19]. The interactions among the five bones were defined as contact surfaces to inhibit intersection, which allowed relative articulating movement. To simulate the contact behavior between bones at articular surfaces, interference detection was applied in Simulation in the joint space in the first place to exclude bone overlap because the surface of the bone would produce small changes during the use of curve- and surface-fitting techniques during modeling, whereas overlap could be present in the bone complex reassembled according to the initial position, leading to computational errors. The contact options were set as no penetration and surface-to-surface. To simulate the lubricating nature of the articular cartilage surfaces, the contact behavior between the articular surfaces was considered as frictionless. The effects of gravity were also considered as negligible in the model.

In the models, one or more linear, tension-only springs were used depending on their geometries to simulate ligament connections, which passed directly between insertions. Attachment points on the bones were identified based on anatomical atlases [20]–[21], published studies (Interactive foot and ankle, Primal Pictures Limited, London, UK), and dissection. A total of 31 springs were included and defined by connecting the corresponding attachment points to simulate connected structures, such as periarticular ligaments and the crural interosseous membrane. Initial ligament lengths were taken as the distance between insertion sites in the neutral position. Some ligaments with a relatively large diameter-to-length ratio were represented by multiple elements according to anatomical knowledge and the literature [22] so as to simulate the recruitment of different ligament fibers under different loading conditions, such as anterior tibiofibular (2 springs), posterior tibiofibular (2 springs), dorsal talonavicular (2 springs), interosseous talocalcaneal (2 springs), plantar calcaneonavicular (2 springs), posterior talofibular (2 springs), tibiocalcaneal (2 springs), and posterior tibotalar ligament (4 springs).

After taking into account that all ligaments possess various anatomic characteristics and mechanical properties, the material properties of each ligament were determined by extensive reviews of existing literature. Data by Siegle et al. [23] were consulted for the medial and lateral collateral ligaments of the ankle and the interosseous talocalcaneal ligaments. The distal tibiofibular syndesmosis ligaments referred to the experimental data of Hoefnagels et al. [24] and Beumer et al. [25]. The other ligaments whose data were not found in literature or varied greatly were all assumed to have a stiffness value between 70 and 90 N/mm [26]. The crural interosseous membrane was represented by four springs, each with a stiffness of 400 N/mm, which was selected according to the lengths of the tibia and fibula, along with stiffness data found on the interosseous membrane of the forearm [27]. To apply the methodology used by Liacouras and Wayne [26], a prestretch (implemented by a reduction in zero-load length of 2%) was applied to each ligament to represent in situ levels, thereby preloading the ligaments, and a prestretch value of 0.5% was applied for the springs representing the interosseous membrane.

The ITSI model was simulated by suppressing the springs representing the anterior and posterior tibiofibular ligaments, the interosseous tibiofibular ligament, along with 8 cm of the distal interosseous membrane closest to the tibiotalar joint. For the final simulation, the fixation model with screw was established based on the injury model described above. Given the complex geometry of the screw, it was assumed as a simple cylinder, and the threads were ignored. At the plane 2.5 cm above the ankle mortise a 3.5-mm hole was generated parallel to the ankle articular surface and through the center of the fibular and tibial diaphyses; the cylindrical surfaces of the holes were then connected with a 316 stainless steel pin on both sides. Two ends of the cylinder were fixed on the fibula and tibia to simulate the effect of the threaded section so that neither sliding nor rotation was allowed between the corresponding contact surfaces. The Young's modulus and Poisson's ratio of the screw were separately defined as 1.93×10^5^ MPa and 0.27.

Three loadings at neutral position with single-foot standing and internal and external ankle rotations were simulated in all three models. The calcaneus and navicular were fixed in space, and the central points of the upper sections of tibia and fibula were fixed in the X and Y directions at the reference surface in the anterior view to make the model stable while the remaining bones were free to move. For a subject with body mass of 60 kg, a 600-N vertical force was applied on the upper section of the tibia to simulate balanced single-foot standing to bear the entire body's weight. Then the constraints remained unchanged, and both a 15 lbf (67 N) of compression load and a 2.7 N • m (24 in. lb) of clockwise or counterclockwise torsional force was applied at the proximal tibia to simulate internal and external rotation of the ankle, with the longitudinal axis of the tibia set as the rotation axis.

After these boundary conditions were set, the models were meshed with second-order 10-node tetrahedral elements using a high-quality grid generation tool in Simulation. Grid density was set to “good,” grid parameters to “normal grid,” overall size to 2.82339 mm, tolerance to 0.14117 mm, and node of Jacobi to 4. Finally, iteration computation was run with the Simulation automatic solver for static solution. All models mentioned above were in complete parallel to carry out more accurate evaluations of relative differences between models.

In this study, in addition to gaining Von Mises stress and resultant displacement distribution and contact force of articulations from the direct calculation results, we also measured the maximum displacement of the tibia and the fibula at the plane 1 cm proximal to the tibia plafond in the anterior/posterior and medial/lateral directions according to common clinically used methods [28]. In the meantime, the magnitude of ligament elongation was also calculated by extracting the 3D coordinates of their attachment points on the bone according to the following formulas:
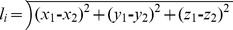
Where:

l_i_ is the initial length of the ligament, x_1_, y_1_, and z_1_ are 3D coordinate components of one attachment point of the ligament, and x_2_, y_2_, and z_2_ are 3D coordinate components of another attachment point of the ligament.
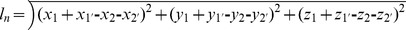
Where:

l_n_ is the new length of the ligament after loading; x_1′_, y_1′_, and z_1′_ are three displacement components of one attachment point of the ligament in the X, Y, and Z directions; and x_2′_, y_2′_, and z_2′_ are three displacement components of another attachment point of the ligament in the respective direction.

So:
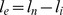
Where:

l_e_ is the magnitude of elongation of the ligament.

Then, through the magnitude of elongation of the ligament, the magnitude of load experienced was inferred from its stiffness and prestretch values.

## Results

**Figure 1:**
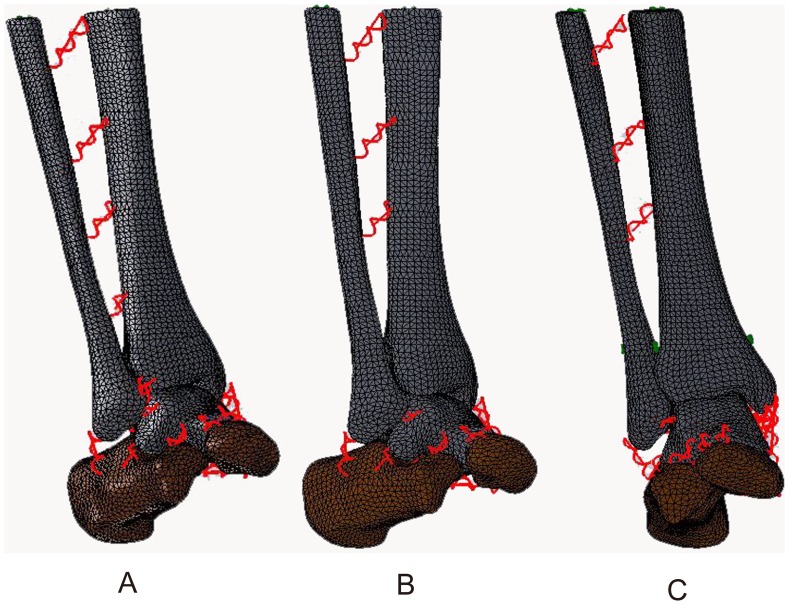
Three 3D FE models were established. A: The normal ankle; B: The tibiofibular syndesmosis injury; C: The screw fixation.

Three models were established of the normal ankle, ITSI, and screw fixation (Figure 1 A, B, C). The total number of elements in each model ranged from 59,651 to 63,299 and nodes ranged from 91,869 to 96,966. Altogether, nine loadings were conducted for the three models.

### Model validation

**Figure 2:**
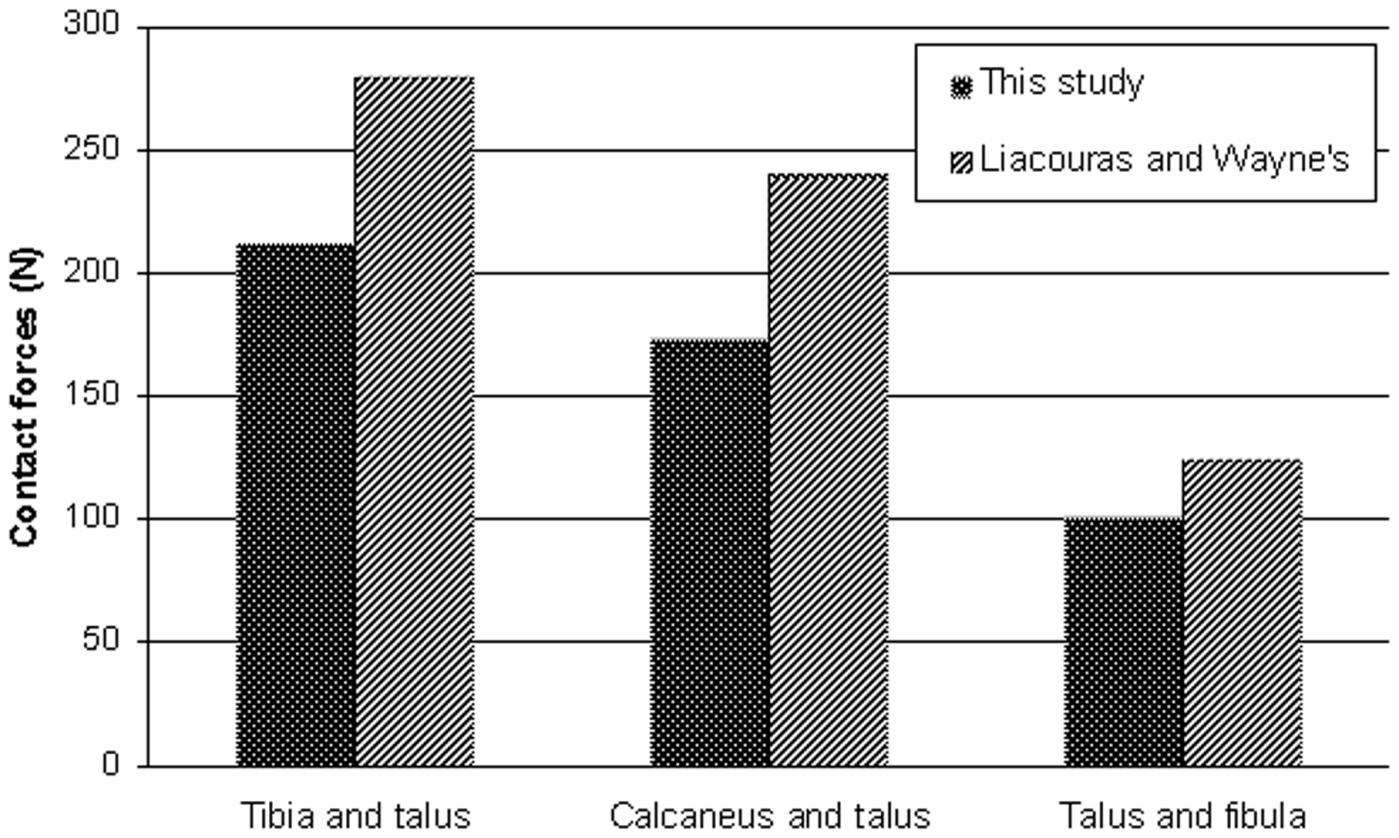
Comparison of present study and published results. Comparison of the magnitudes of contact forces seen in the major articulations after application of external rotation load between this study and Liacouras and Wayne's.

**Figure 3:**
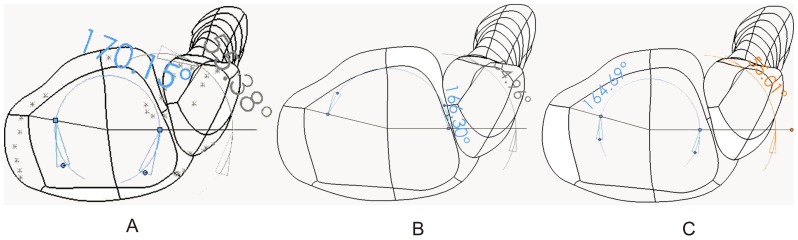
Angles measured between the horizontal and straight lines of the tibia and fibula on the ankle plane in our models. A: Before loading; B: During external rotation of the normal ankle; C: During external rotation of the ITSI.

Because the boundary conditions of this study were based on the research methods used by Liacouras and Wayne [26], it was sensible to compare our results with theirs to validate the models. In the simulation of loading during external rotation of the ankle, our results were in agreement with theirs regarding the distribution of contact forces between the major articulations in the intact ankle model; both displayed a trend of the contact forces between the talus and tibia > those between calcaneus and talus > those between talus and fibula and similar magnitudes of forces (Figure 2). Meanwhile, the internal rotation angles of the tibia in the intact ankle were 3.85° and 4.28°, respectively, but after cutting the syndesmosis they increased to 5.46° and 5.60°, respectively, for the present study (Figure 3 A, B, C) and Liacouras and Wayne's [26]. Although they did not simulate other loading methods, the results of our model in simulating internal rotation of the ankle and neutral position with single-foot standing were considered reliable.

### Von Mises stress distribution

**Figure 4:**
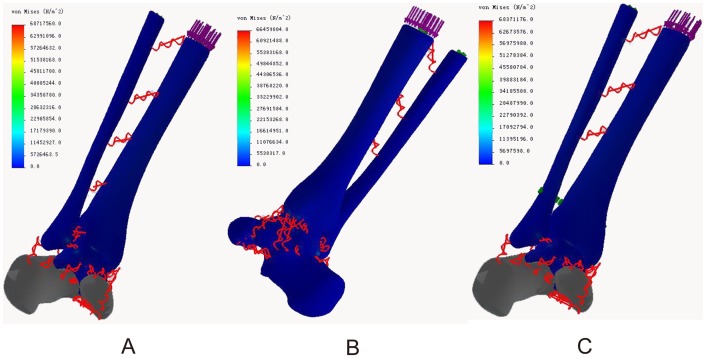
Von Mises stress distribution in three models during neutral position with single-foot standing. A: The normal ankle; B: The tibiofibular syndesmosis injury; C: The screw fixation.

In the neutral position, the maximum stresses of all three models were located in the middle of the posterior talocalcaneal joint surface of the talus (68.72, 66.46, and 68.37 Mpa, respectively). The ligament attachment points had acceptable stress and minimal differences. The maximum ligament stresses were located at the talus attachment point of the posterior talocalcaneal ligament in models of both normal ankle (11.88 Mpa) and ITSI (10.67 Mpa) and at the talus attachment point of anterior tibiotalar ligament in the screw fixation model (12.04 Mpa). The maximum stresses of the ankle joint surface of the talus were 44.94 Mpa, 45.2, and 42.54 Mpa in three models, respectively (Figure 4 A, B, C).

**Figure 5:**
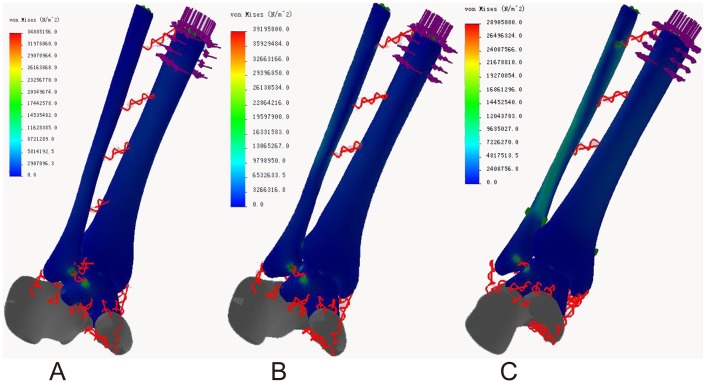
Von Mises stress distribution in three models during internal rotation of the ankle. A: The normal ankle; B: The tibiofibular syndesmosis injury; C: The screw fixation.

For internal rotation of the ankle, the maximum stresses in the normal ankle (34.89 Mpa) and ITSI (39.20 MPa) models were located at the fibula attachment point of the anterior talofibular ligament and at the fibula attachment point of the proximal crural interosseous membrane in the screw fixation model (28.91 Mpa). The maximum stresses of the ankle joint surface of the talus were 11.27, 13.92, and 10.4 Mpa in three models, respectively (Figure 5 A, B, C).

**Figure 6:**
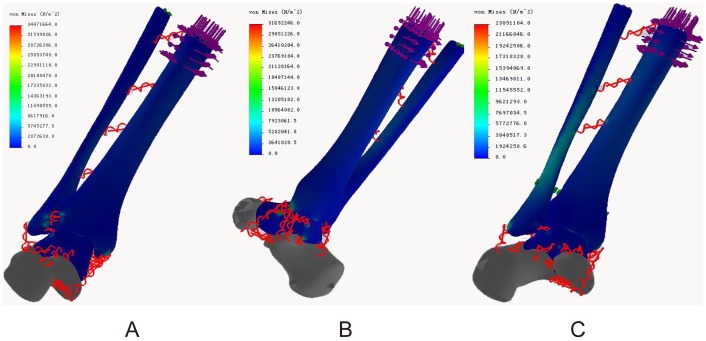
Von Mises stress distribution in three models during external rotation of the ankle. A: The normal ankle; B: The tibiofibular syndesmosis injury; C: The screw fixation.

In the external rotation of the ankle, the maximum stresses in the normal ankle (34.47 Mpa) and screw fixation (23.09 Mpa) models were located in the middle of the posterior talocalcaneal joint surface of the talus and in the middle of the posterior ankle joint surface of the talus in the ITSI model (31.69 Mpa). The maximum ligament stresses were located at the talus neck attachment point of the anterior tibiotalar ligament in models of both normal ankle (14.66 Mpa) and ITSI (19.61 Mpa) and at the talus attachment points of the medial talocalcaneal ligament in the screw fixation model (12.96 Mpa). The maximum stresses of the ankle joint surface of the talus were 22.68, 31.69, and 17.53 Mpa in three models, respectively (Figure 6 A, B, C).

Moreover, the stresses on the fibula were both increased around and above the screw during internal and external rotation simulations of the ankle.

### Resultant displacement distribution

**Figure 7:**
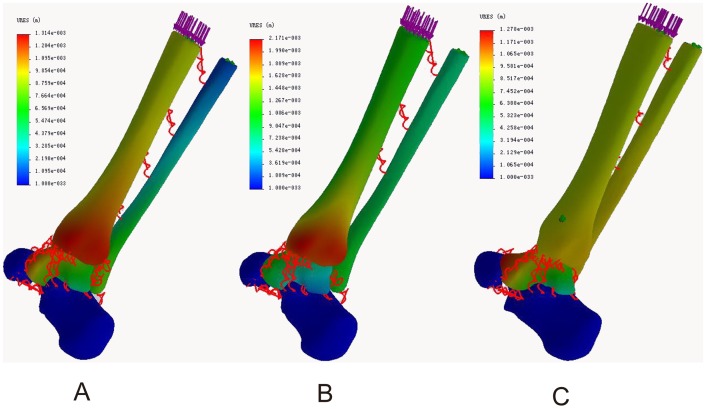
Resultant displacement distribution in three models during neutral position with single-foot standing. A: The normal ankle; B: The tibiofibular syndesmosis injury; C: The screw fixation.

In the neutral position, the maximum displacements of the tibia were located in the medial posterior part of the medial malleolus in both normal ankle (1.31 mm) and ITSI (2.17 mm) models and located in the posterior part of the posterior malleolus in the screw fixation model (1.02 mm). The maximum fibula displacements were located in the medial posterior part of the lateral malleolus in the model of normal ankle (0.80 mm), the medial part of the distal fibula in the ITSI model (1.03 mm), and the lateral part of the distal fibula in the screw fixation model (1.01 mm). The maximum displacements of the talus were located in the anterior part of the talus head in the normal ankle model (1.29 mm), the superior lateral part of the talus head in the ITSI model (1.32 mm), and the superior anterior part of the talus head in the screw fixation model (1.28 mm) (Figure 7 A, B, C).

**Figure 8:**
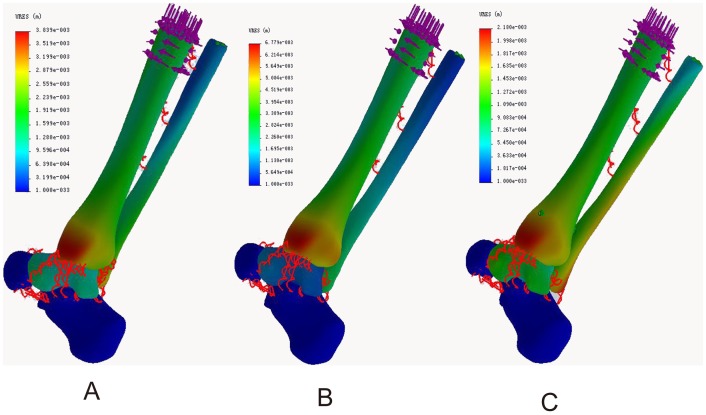
Resultant displacement distribution in three models during internal rotation of the ankle. A: The normal ankle; B: The tibiofibular syndesmosis injury; C: The screw fixation.

During internal rotation of the ankle, the maximum tibial displacements were located in the medial part of the medial malleolus in all three models (3.84, 6.78, and 2.18 mm). The maximum fibular displacements were located in the lateral part of the tip of the lateral malleolus in the normal ankle (3.25 mm) and screw fixation (2.14 mm) models and in the middle of the talofibular articular surface in the ITSI model (2.88 mm). The maximum talus displacements were located in the anterior of the talus head in models of both normal ankle (1.45 mm) and ITSI (1.22 mm) and in the anterior medial part of the talus head in the screw fixation model (1.27 mm) (Figure 8 A, B, C).

**Figure 9:**
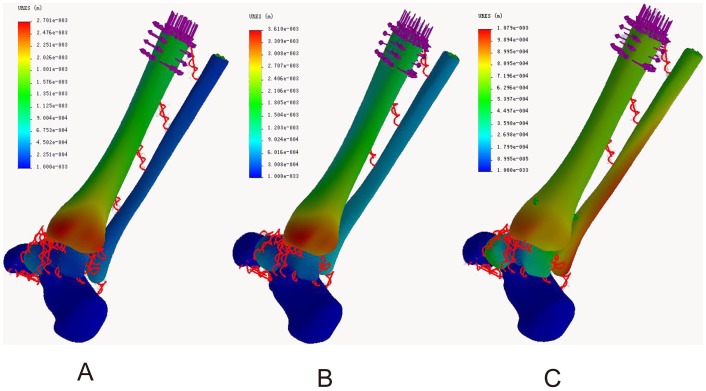
Resultant displacement distribution in three models during external rotation of the ankle. A: The normal ankle; B: The tibiofibular syndesmosis injury; C: The screw fixation.

During external rotation of the ankle, the maximum tibial displacements were located in the medial part of the medial malleolus in all three models (2.70, 3.61, and 0.97 mm). The maximum fibular displacements were located in the lateral part of the tip of lateral malleolus in all three models (0.52, 0.87, and 1.08 mm). The maximum displacements of the talus were located in the anterior lateral part of the talus head in the model of normal ankle (0.71 mm), in the superior lateral part of the talus body in the ITSI model (0.91 mm), and in the superior lateral part of the talus head in the screw fixation model (0.82 mm) (Figure 9 A, B, C).

### The magnitude of contact forces in the major articulations

**Figure 10:**
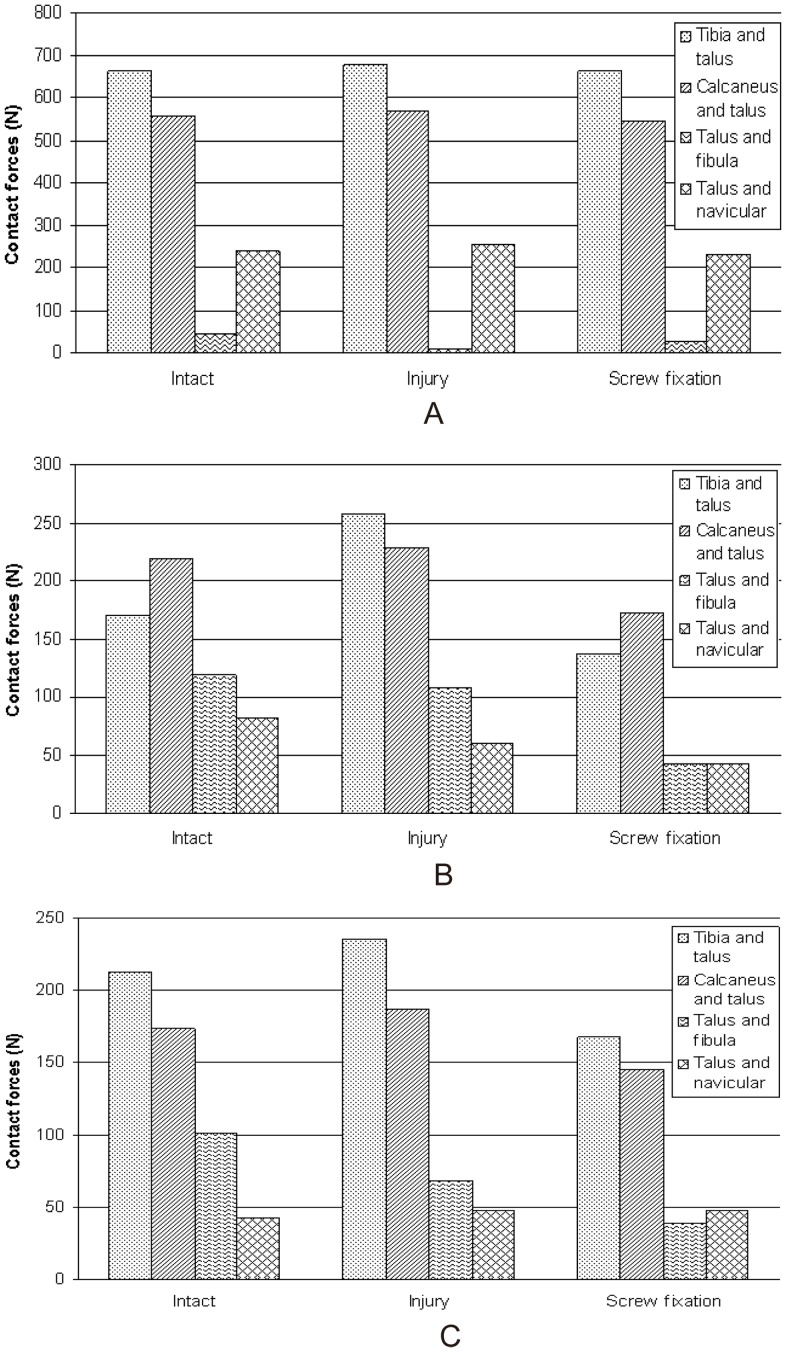
The magnitude of contact forces seen in the major articulations after application of three loads. A: Neutral position with single-foot standing simulation; B: Internal rotation of the ankle simulation; C: External rotation of the ankle simulation.

Contact forces are those that occur between contacting objects. The results (Figure 10 A, B, C) demonstrated that compared with the normal model, ITSI reduced contact forces between the talus and fibula, especially in the neutral position with single-foot standing and during external ankle rotation; whereas contact forces of the tibiotalar, talocalcaneal, and talonavicular joints mainly exhibited mild increases in all three loading states. However, contact forces of all these hindfoot joints were reduced to some degree after screw fixation.

### Magnitude of elongation and load in the major springs (ligaments) resisting movement

Due to tibiofibular syndesmosis ligament dysfunction, the stress distribution of other ligaments around the ankle changed, which increased the stress on some ligaments during various ankle motions (Tables 1, Tables 2, and Tables 3). For example, in the neutral position with single-foot standing, stress on the lateral malleolus ligaments was reduced, while stress on the anterior part of the posterior tibiotalar ligament of the medial malleolus was increased. When the ankle was internally rotated, the magnitude of load experienced in the anterior talofibular ligament was increased from 133 N to 171 N, and that in the posterior part of the posterior tibiotalar ligament of the medial malleolus increased from 37 N to 155 N. During external rotation, the magnitude of load experienced in the anterior tibiotalar ligament increased from 86 N to 123 N.

**Table 1:** Magnitude of elongation (mm) and load (N) seen in the major ligaments resisting movement in three models during neutral position with single-foot standing load.

	Intact		Injury		Screw fixation	
Ligaments	Elongation	Load	Elongation	Load	Elongation	Load
Anterior talofibular	0.01	32.74	−0.19	3.37	−0.03	27.16
Posterior talocalcaneal	0.38	40.06	0.32	35.58	0.34	37.11
Anterior tibiotalar	0.05	39.56	−0.15	16.01	0.17	55.03

As to the effect of stress on periarticular soft tissue after screw fixation, we found that the magnitude of load experienced in the anterior tibiotalar ligament from 40 N to 55 N in the neutral single-foot standing position. Conversely, ligaments around the ankle exhibited lower stress than the normal model during the two torsional loads, and stress on the crural interosseous membrane increased. For example, during internal rotation of the ankle, the magnitude of load experienced in the proximal crural interosseous membrane increased from 16 N to 90 N; when the ankle was externally rotated, the magnitude of load experienced in the distal crural interosseous membrane increased from 0 N to 27 N.

**Table:** Table 2. Magnitude of elongation (mm) and load (N) seen in the major ligaments resisting movement in three models after application of internal rotation load.

	Intact		Injury		Screw fixation	
Ligaments	Elongation	Load	Elongation	Load	Elongation	Load
Posterior tibiofibular (proximal)	0.13	37.26	–	–	–	–
Posterior tibiofibular (distal)	0.12	51.90	–	–	–	–
Anterior talofibular	0.72	133.03	0.99	171.32	0.33	77.93
Lateral talocalcaneal	0.01	21.53	−0.09	13.89	−0.10	13.11
Posterior talocalcaneal	0.49	49.56	0.36	38.76	0.41	43.10
Posterior tibotalar(pos. 1)	0.01	14.88	1.09	79.20	−0.12	6.53
Posterior tibotalar(pos. 2)	0.16	22.64	1.04	75.58	−0.12	5.85
Interosseous talocalcaneal (posterior)	0.11	57.18	−0.02	26.32	0.01	32.51

**Table:** Table 3. Magnitude of elongation (mm) and load (N) seen in the major ligaments resisting movement in three models after application of external rotation load.

	Intact		Injury		Screw fixation	
Ligaments	Elongation	Load	Elongation	Load	Elongation	Load
Anterior tibiofibular (proximal)	0.19	41.03	–	–	–	–
Anterior tibiofibular (distal)	0.08	27.07	–	–	–	–
Anterior talofibular	0.00	31.20	0.11	46.95	−0.16	8.71
Calcaneofibular	0.07	59.07	−0.08	39.36	0.06	57.72
Anterior tibiotalar	0.42	85.63	0.73	123.01	0.04	39.04
Medial talocalcaneal	0.15	27.86	0.28	38.76	0.09	23.06
Posterior talocalcaneal	0.01	10.70	−0.05	5.84	−0.01	8.84

### Translations of the fibula and tibia

**Figure 11:**
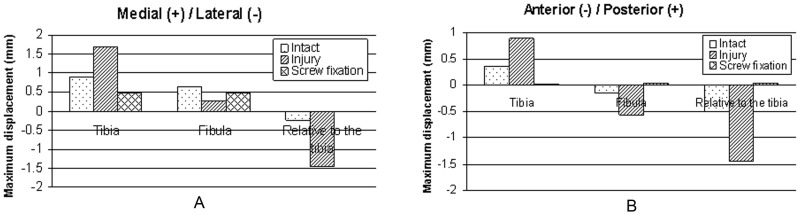
Displacements of the tibia and the fibula during neutral position with single-foot standing. Maximal displacements and relative displacements at the plane 1/posterior (A) and medial/lateral directions (B).

**Figure 12:**
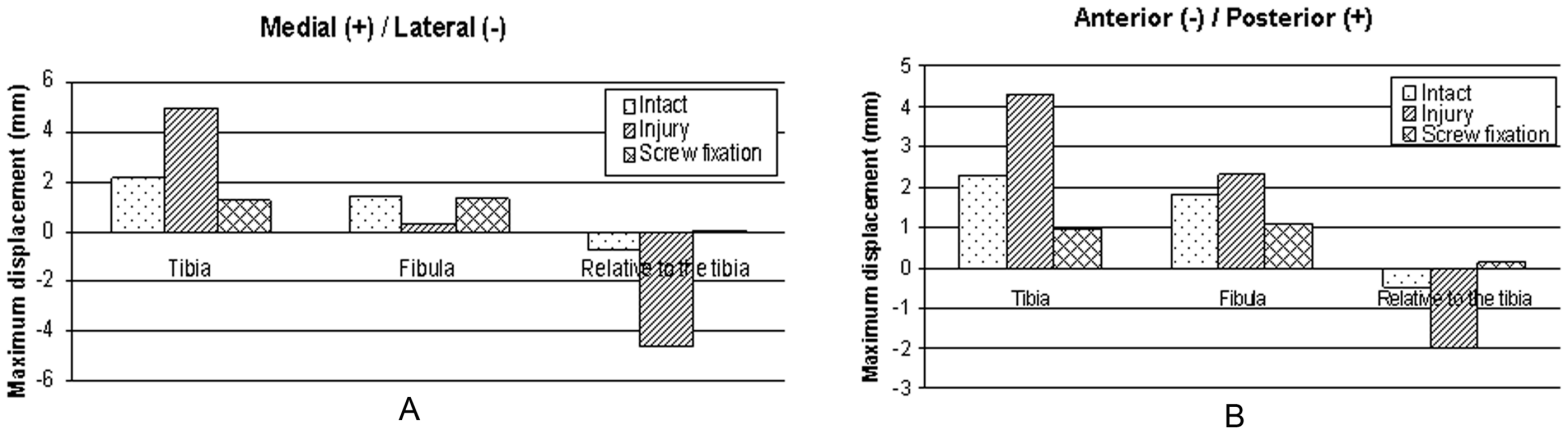
Displacements of the tibia and the fibula during internal rotation of the ankle. Maximal displacements and relative displacements at the plane 1/posterior (A) and medial/lateral directions (B).

**Figure 13:**
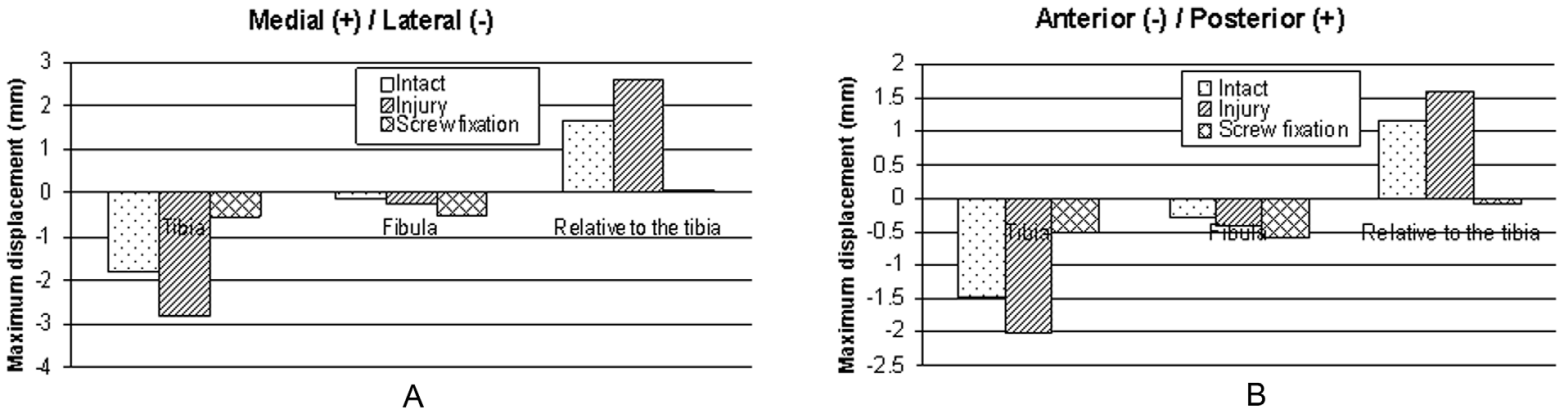
Displacements of the tibia and the fibula during external rotation of the ankle. Maximal displacements and relative displacements at the plane 1/posterior (A) and medial/lateral directions (B).

The maximum displacement values were compared among the three models. The figures (Figure 11 A, B, Figure 12 A, B, Figure 13 A, B) demonstrate that in contrast to the normal ankle, the translations of the fibula relative to the tibia increased significantly in the anterior/posterior and medial/lateral directions after tibiofibular syndesmosis injury. The maximum translations were 4.63 mm in the medial/lateral directions and 2.01 mm in the anterior/posterior directions during loading for internal ankle rotation. The translations were significantly reduced in various directions after syndesmosis screw fixation.

## Discussion

The syndesmotic ligament complex consists of the anterior inferior tibiofibular ligament (AITFL), posterior inferior tibiofibular ligament (PITFL), and interosseous ligament (IL) [3]. The inferior transverse tibiofibular ligament is sometimes considered a fourth ligament but is really a continuation of the distal PITFL rather than a separate structure. Collectively, these ligaments prevent joint diastasis.

An isolated syndesmotic injury is often referred to as a “high ankle sprain.” Syndesmotic injuries are graded from I to III in the same manner as lateral ankle sprains. Grade I refers to a mild stretching of the syndesmotic ligaments, grade II injuries represent an incomplete tear of the syndesmosis, and a grade III sprain is a complete disruption of the syndesmosis [29]. In this study, the injury model was equivalent to a grade III sprain. The influences of various degrees of syndesmotic injury on the ankle will be explored in future studies.

Our results show that the stress and displacement distribution of the ankle changed greatly after ITSI. The normal force transmission relationship of the fibula was reduced, which resulted in increased contact force of the tibiotalar joint; conversely, contact forces between the talus and fibula decreased, especially in the state of neutral position with single-foot standing, which was equivalent to one-sixth the magnitude of the normal model. The study by Liacouras and Wayne [26] also confirmed that contact forces between the talus and fibula significantly decreased during their simulation of external ankle rotation but found no difference in tibiotalar joint and talocalcaneal joint contact forces. It also showed that several ligaments displayed more resistance than others with a large resulting force during internal and external ankle rotation. For example, the anterior talofibular and posterior talocalcaneal ligaments experienced larger forces in all three loading simulations. These ligaments and the bony architecture are rotational/translational restricting factors. Liacouras and Wayne [26] found that the calcaneofibular ligament suffered the most loading in two of the three simulations of their load simulation of external ankle rotation, but our study showed that the anterior tibiotalar ligament experienced the most loading in intact and injury models during this load simulation. In our study of ITSI, the loads experienced in the medial collateral ligaments of ankle, such as the anterior tibiotalar ligament and the posterior tibiotalar ligament, were significantly increased during simulations of internal and external ankle rotation in contrast to the intact ankle configuration. It reflected that ITSI would cause the magnitude of the load to resist movement and increase stress on medial malleolus ligaments, which increases the risk of medial collateral ligaments injury or medial malleolar fracture. Therefore, it is logical that clinical ITSI often occurs in tandem with medial malleolus fracture or medial collateral ligament injuries, which can be seen in Lauge-Hansen classification's pronation-external rotation IV degree and fibula fracture above the joint space in the supination-external rotation ankle fracture-dislocation; IV degree fractures are often accompanied by tibiofibular syndesmosis diastasis.

**Figure 14:**
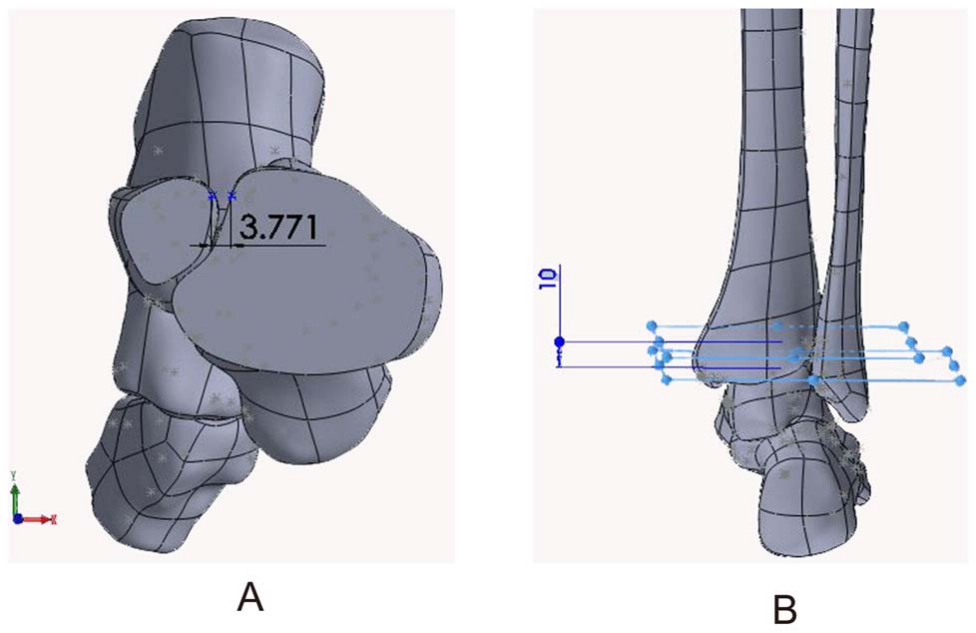
The tibiofibular clear space of unloading model measured in this study. The distance between the lateral border of the posterior tibial tubercle and the medial border of the fibula (A) 10 mm proximal to the distal tibial articular surface (B).

Tibiofibular syndesmosis injuries usually display relative tibia and fibula displacements in various orientations. X-rays are usually used to diagnose tibiofibular syndesmosis injuries in clinical practice. Three parameters are utilized to evaluate syndesmotic injuries: tibiofibular clear space (TFCS), medial clear space, and tibiofibular overlap. TFCS is the most reliable of these indicators, as it is not significantly influenced by tibial rotation [30], which is defined as the distance between the lateral border of the posterior tibial tubercle and the medial border of the fibula and is measured on anterior/posterior and mortise radiographs 1 cm proximal to the distal tibial articular surface. A distance of less than approximately 6 mm measured on anterior/posterior and mortise views seems normal [28]. However, this approach has some limitations. For example, experiential observation or manual measurement with a ruler cannot accurately describe the displacement distance in space. Moreover, projection position and imaging quality also influence the result. Digital technology provides great convenience to provide the exact values by detecting the corresponding nodes of the model, which is why we measured displacements in our FE models. The value of the unloading model was approximately 3.77 mm in our study (Figure 14 A, B), which is similar with previously described CT measurements [31].

Our findings show that tibiofibular syndesmosis injury would lead to increased movement of the distal tibia and fibula. The anterior/posterior and medial/lateral displacements of the tibia and fibula increased in the coronal and sagittal planes of the human body during the three loading conditions. However, although the absolute distances in the inferior tibiofibular joint were widened during loading at standing in the neutral position and internal rotation of the ankle, the clear space in the joint approached and exceeded the previously proposed 6-mm criterion [28]. Besides, the absolute distances were narrowed during loading when the ankle was externally rotated. This may be explained by differences in research methods. Furthermore, the results also indicated the TFSC was associated with the loading state at the foot and ankle, which should be clinically recognized.

Most syndesmosis injuries without mortise widening are minor and are thus treated conservatively, though the recovery period is often prolonged. Fixation of the distal fibula to the tibia is the preferred treatment for severe syndesmosis sprains as studied here because it leads to anatomical reduction and healing and allows patients to resume weight-bearing activities. There are many methods to stabilize the disrupted syndesmosis, but the most often used is that recommended by AO to place a fully threaded transverse syndesmotic screw because the effectiveness of tibiofibular syndesmosis width variation under screw control has been repeatedly confirmed. It has been advised that the screw should be positioned between 2 and 3 cm proximal to the tibial plafond [32], but there is no general agreement as to which injuries benefit from internal fixation with a syndesmotic screw [33]. So far, no consensus has been achieved regarding the optimal number of cortices, screw size, position of the foot during screw insertion, use of one or two screws, position of the screw(s) relative to the tibiotalar joint, use of bioabsorbable screws, time to weight bearing, or removal or not of the screw before weight bearing [34].

Although some controversies still exist [35]–[38], there is general consensus on some aspects, such as the number of cortices and screw material and diameter, which have a relatively small impact in the short term. Taking into account the feasibility and the main purpose of the digital models we built, as well as actual clinical conditions, our digital models explored the biomechanical effects of screw fixation on the ankle using 3.5-mm 316 stainless steel screws in fixation through the center of tibia and fibula.

Our results demonstrate that after screw fixation, except for slightly increased contact forces between the talus and tibia in the neutral position, the other hindfoot joint forces were all decreased. At the same time, stresses on ligaments around the ankle were also decreased; however the stresses on the crural interosseous membrane were distinctly increased after torsional load application. In addition, movements of the distal tibia and fibula were significantly decreased in the anterior/posterior and medial/lateral directions after fixation with a syndesmosis screw. The absolute displacements were both less than 0.3 mm between the distal tibia and fibula.

In conclusion, severe syndesmosis injuries will greatly change ankle stress distribution and cause multidirectional ankle instability. Therefore, reduction and operative stabilization are necessary. Ideally, the implant should both stabilize the syndesmosis and allow physiologic micromotion and early mobilization, but it is not easy to achieve these goals in the clinic. Effective fixation for a period of time can compromise the measure to promote scar healing of the syndesmosis. Although a transverse syndesmotic screw can effectively control excessive abnormal activity of the distal tibia and fibula following tibiofibular syndesmosis injury, screw fixation also affects the physiologic normality of the joint, leading to decreased magnitude of motion at the lower extremes of the tibia and fibula, reduced contact forces between bones, and increased stress on the crural interosseous membrane. Moore et al. [39] stated that retention of syndesmotic screws, even with mechanical failure, did not pose a clinical problem, and weight bearing could be allowed at 6 to 10 weeks without routine screw removal. However, considering that the ultimate goal of treatment is to restore joint function, we support that screws should be removed once healing is achieved in order to restore normal function and the stress transfer mechanism of the ankle joint as soon as possible.

Difficulties in determining typical values for the mechanical characterization of materials are evident when dealing with human tissues. In addition, experimental studies have provided limited data regarding ankle joint characteristics that can be used in FE models. Moreover, because a large number of nonlinear problems are involved in the modeling and calculation, the convergence of results is not easy to control. Taking into the situation into account in most cases, bone was considered homogeneous, isotropic, and linearly elastic. Ligaments were represented as linear springs in an attempt to replicate the behavior associated with the linear region of ligament tensile behavior. Articular cartilage deformation was neglected because the bones were represented by rigid objects; cartilage function was incorporated by neglecting friction in the model. Because it is difficult to obtain the actual prestress on ligaments, this study consulted the methodology employed by Liacouras and Wayne [26]; however, recent data indicate that the difference between in situ and ex vivo measurements can be as high as four orders of magnitudes [40]. These and other limitations of the models, such as the simplified screw geometry, must be taken into account when considering a direct transfer of the results presented here into the clinical situation. However, the main concern of this research was the relative differences between the models studied rather than a quantitative assessment of biomechanical indicators in each specific case. Any inaccuracies introduced would be present in all models, which had little effect on this comparative study.

Present research methodologies only can perform approximate simulations that do not fully mimic the clinical situation. For example, we eliminated secondary factors that do not strongly influence the results. If proper material parameters and boundary conditions are selected, the results will be clinically significant. Our models show the advantages of digital techniques in acquiring extensive and numerically precise data, which are not easy to achieve by traditional biomechanical experiments. Overall, the findings of the present study are helpful in guiding the clinical diagnosis and management of tibiofibular syndesmosis injuries. We will further verify the results in subsequent clinical studies.
